# Lentivirus-Mediated Nox4 shRNA Invasion and Angiogenesis and Enhances Radiosensitivity in Human Glioblastoma

**DOI:** 10.1155/2014/581732

**Published:** 2014-04-27

**Authors:** Yongsheng Li, Na Han, Tiejun Yin, Lulu Huang, Shunfang Liu, Dongbo Liu, Cuihong Xie, Mengxian Zhang

**Affiliations:** ^1^Department of Emergency Medicine, Tongji Hospital, Tongji Medical College, Huazhong University of Science & Technology, Wuhan 430030, China; ^2^Department of Oncology, Tongji Hospital, Tongji Medical College, Huazhong University of Science & Technology, Jiefang Road 1095, Wuhan 430030, China

## Abstract

Radioresistance remains a significant therapeutic obstacle in glioblastoma. Reactive oxygen species (ROS) are associated with multiple cellular functions such as cell proliferation and apoptosis. Nox4 NADPH oxidase is abundantly expressed and has proven to be a major source of ROS production in glioblastoma. Here we investigated the effects of Nox4 on GBM tumor cell invasion, angiogenesis, and radiosensitivity. A lentiviral shRNA vector was utilized to stably knockdown Nox4 in U87MG and U251 glioblastoma cells. ROS production was measured by flow cytometry using the fluorescent probe DCFH-DA. Radiosensitivity was evaluated by clonogenic assay and survival curve was generated. Cell proliferation activity was assessed by a cell counting proliferation assay and invasion/migration potential by Matrigel invasion assay. Tube-like structure formation assay was used to evaluate angiogenesis ability *in vitro* and VEGF expression was assessed by MTT assay. Nox4 knockdown reduced ROS production significantly and suppressed glioblastoma cells proliferation and invasion and tumor associated angiogenesis and increased their radiosensitivity *in vitro*. Our results indicate that Nox4 may play a crucial role in tumor invasion, angiogenesis, and radioresistance in glioblastoma. Inhibition of Nox4 by lentivirus-mediated shRNA could be a strategy to overcome radioresistance and then improve its therapeutic efficacy for glioblastoma.

## 1. Introduction

Glioblastoma multiforme (GBM) is the most common and most malignant primary brain tumor in adults with a high degree of morbidity and mortality [1]. Recent advances in the treatment of glioblastoma multiforme published in the landmark study by Stupp et al. [2] changed the standard of care with the discovery that the addition of temozolomide (TMZ) to radiotherapy for newly diagnosed glioblastoma resulted in a clinically meaningful and statistically significant survival benefit with minimal additional toxicity. Despite this success, survival remains very low. Virtually all patients suffer tumor recurrence despite aggressive irradiation, emphasizing the radioresistant nature of GBMs and prompting investigators to seek alternate treatments through a better understanding of the cell biology and through some new molecular targets that may enhance current treatments.

Reactive oxygen species (ROS) are highly reactive O_2_ metabolites that include superoxide anion (O_2_^−^), hydrogen peroxide (H_2_O_2_), and hydroxyl radical (OH^−^). Although ROS are classically thought of as cytotoxic and mutagenic, recent evidence suggests that ROS serve as regulators of signal-transduction pathways for cell proliferation and survival [3]. Nox-family NADPH oxidases have proven to be a major source of ROS production in various cell types and have crucial roles in various physiological and pathological processes [4]. Several studies demonstrated that NADPH oxidase subunit 4 (Nox4) is expressed in several human tumors, such as glioblastoma [5], hepatocellular carcinoma [6], breast cancer [7], thyroid cancer [8], and melanoma [9], and is involved in cellular senescence, resistance to apoptosis, tumorigenic transformation, cell proliferation, cell survival, and chemotherapy resistance. Strong evidence suggests that these processes are upregulated via Nox4 generation of ROS.

In glioblastoma, the expression levels of Nox4 mRNA were significantly higher than those in other astrocytomas (WHO grades II and III). Specific knockdown of Nox4 expression by RNA interference resulted in cell-growth inhibition and enhanced induction of apoptosis by chemotherapeutic agents [5], indicating that enhanced expression of Nox4 appears to be involved in cell proliferation and chemotherapy resistance in glioma cells. Most recent studies have shown that Nox4 played important roles in cycling hypoxia-mediated HIF-1 activation and further promoted tumor progression in glioblastoma [10, 11], suggesting that Nox4 might be a critical mediator of radioresistance in GBM, since hypoxia is known as a major reason for the resistance of tumor cells to radiation [12]. Lu et al. have shown that androgens increased ROS production by Nox4 and Nox2 in prostate cancer. Preradiation treatment of human prostate cancer cells with Nox inhibitors sensitized the cells to radiation similar to androgen deprivation therapy [13], further supporting the notion that enhanced Nox4 levels are protective against radiation-induced tumor cell death. Moreover, Nox4 has been shown to promote invasion and angiogenesis process in several solid organ tumors, such as renal cell carcinoma [14], ovarian cancer [15], and head and neck cancer [16]. Invasive tumor growth and active neovascularization are characteristic features of GBM, which contribute to its radioresistant phenotype. Based on these data, we hypothesized that Nox4 might be a critical mediator of invasion, angiogenesis, and radiation response in GBM and a potential target for developing better therapeutic methods.

To explore the potential impact of Nox4 on GBM radiosensitivity, invasion, and angiogenesis, a loss-of-function analysis was performed by applying a Nox4 short hairpin RNA- (shRNA-) expressing lentivirus to two GBM cell lines, U87MG and U251. Then the effect of Nox4 knockdown on the colonies formation, cell proliferation, and cell invasion ability of GBM was investigated. Moreover, capillary tube-like structure formation ability of human umbilical veins endothelial cells (HUVECs) that were cocultured with conditioned medium derived from GBM cells was measured. Our study demonstrates that Nox4 was involved in ROS generation and Nox4 knockdown inhibited cell invasion, angiogenesis, and promoted radiation response in human glioblastoma.

## 2. Methods and Materials

### 2.1. Cell Cultures and Treatment Conditions

Human glioblastoma (U87MG and U251) tumor cells were obtained from the American Type Culture Collection (ATCC; Manassas, VA) and were cultured in DMEM medium supplemented with 10% FCS, 50 mg/mL penicillin/streptomycin. Primary isolated human umbilical vein endothelial cells (HUVEC, Promocell) were cultured up to passage 8. Cells were maintained in culture at 37°C with 5% CO_2_ and 95% humidity in serum reduced (5% FCS) modified Promocell medium (MPM) supplemented with 2 ng/mL VEGF, 4 ng/mL bFGF. For X-ray treatment, cells were cultured in 25 cm^2^ flasks and then irradiated at 2.5 Gy/min, at room temperature, with an Elekta Precise Linear Accelerator operating at 6 MV.

### 2.2. Lentivirus-Mediated shRNA Knockdown of Gene Expression

pGIPZ-lentiviral shRNAmir vectors targeting human Nox4 gene and nonsilencing pGIPZ control vector were purchased from Open Biosystems (Thermo Fisher Scientific, Inc.). pGIPZ cloning vector contains Turbo GFP reporter and expresses a puromycin-resistant gene. Lentiviral shRNA was produced by cotransfection of the Trans-Lentiviral Packaging Mix with a shRNA transfer vector into HEK 293T packaging cells (Open Biosystems). Supernatants containing either the lentivirus expressing the Nox4 shRNA or the control shRNA were harvested 72 h after transfection. The lentiviruses were purified using ultracentrifugation, and the titer of the lentiviruses was determined. U87MG and U251 cells were transduced by the lentiviral particles at a multiplicity of infection (MOI) of 10 followed by puromycin selection for 10 days. The clones stably transfected with pGIPZ-lentiviral shRNAmir was referred to as Nox4 shRNA cells, whereas the cells stably transfected with pGIPZ nonsilencing control vector as scrambled cells. The knockdown of Nox4 was evaluated by real-time quantitative PCR and Western blot analysis.

### 2.3. Real-Time Quantitative RT-PCR (qRT-PCR)

Total RNA extraction was performed using Trizol reagent (Invitrogen, Carlsbad, CA) according to the manufacturer's instruction and purity and integrity of the RNA was assessed with Agilent 2100 BioAnalyzer (Agilent Technologies, Palo Alto, CA, USA). Then qRT-PCR was performed using QuantiTect Primer Assay (QIAGEN) and QuantiTect SYBR Green RT-PCR Kit (QIAGEN) on a LightCycler 480 Instrument (Roche Diagnostics). The detection and quantification involved the following steps: reverse transcription at 50°C for 30 min, initial activation at 95°C for 15 min, followed by 40 cycles of denaturation at 94°C for 15 sec, annealing at 55°C for 30 sec, and extension at 72°C for 30 sec. Fluorescence data collection was performed at the extension step at 72°C. The relative expression of the target gene was calculated by normalizing the Cp (crossing point) values with those of housekeeping gene GAPDH. All assays were performed in triplicates.

### 2.4. Western Blot Analysis

Protein extracts were prepared by using RIPA lysis buffer and the protein concentrations were measured by the Bradford method using BCA Protein Assay Kit (Pierce Biotechnology, USA). Samples were immunoblotted with antibodies against Nox4 (Cell signalling). An anti-*β*-actin monoclonal antibody purchased from Sigma (St. Louis, MO) was used as an internal loading control. Blots were developed using ECL method and Western band densities were qualified using ImageJ software (National Institutes of Health).

### 2.5. Measurement of the Intracellular ROS Level

Intracellular ROS accumulation was measured by flow cytometry using the fluorescent probe 2′,7′-dichlorodihydrofluorescein diacetate (DCFH-DA, Sigma-Aldrich). Cells were incubated with 10 *μ*mol/L DCFH-DA for 30 min at 37°C in dark. After incubation, the cells were washed with phosphate buffered saline (PBS) and analyzed within 30 min using FACScan flow cytometer (Becton Dickinson, San Jose, CA, U.S.A) with the excitation source at 488 nm and emission wavelength of 525 nm. 10,000 cells were counted in each determination.

### 2.6. Clonogenic Assay

Increasing numbers of cells (10^2^ to 5 × 10^4^) were plated in 25 cm^2^ flasks and irradiated with various doses (0∼8 Gy) with 6 MV X-ray at a dose rate of 2.5 Gy/min. After 10 to 14 days' culture, colonies formed were stained with crystal violet (Sigma) and those with at least 50 cells were counted by microscopic inspection, and plating efficiency as well as clonogenic survival was calculated. The linear-quadratic (LQ) equation was fitted to data sets to generate survival curves and dose enhancement factor (DEF) for drugs was calculated at 10% surviving fraction. DEF values greater than 1.0 indicate enhancement of radiosensitivity.

### 2.7. Proliferation Assay

The effect of Nox4 knockdown on cell proliferation was assessed using a cell counting proliferation assay. In brief, 5 × 10^4^ cells were seeded on 25 cm^2^ flasks over night at standard conditions. The cells were exposed to a single 4 Gy irradiation with 6 MV X-ray and incubated for another 72 h, the cells were harvested and stained with trypan blue, and the total number of living cells was counted by microscopic inspection.

### 2.8. Matrigel Invasion Assays

The invasion of glioblastoma cells* in vitro* was measured on Matrigel-coated (0.78 mg/mL) transwell inserts with 8 *μ*m pore size (BD, Biosciences). The Nox4 shRNA and scrambled cells were starved for 24 hours and then were harvested. 500 *μ*L of cell suspension (1 × 10^5^ cells/mL) per experiment was seeded into the upper well of the chamber containing serum free media. The lower chamber had been filled with DMEM with 10% BSA. After 12 hours of incubation, cells that had invaded the membrane were fixed with 70% ethanol and stained with 0.1% crystal violet and sealed on slides. Representative photos were taken and migrated cells were counted in 6 random high-power fields per chamber under a light microscope.

### 2.9. Tube Formation Assay

To evaluate* in vitro* angiogenesis activity, tube formation assays were performed with HUVEC. 24-well plates were coated with 300 *μ*L Matrigel (BD, Biosciences). HUVECs were suspended in serum-free conditioned medium obtained from culture supernatant of Nox4 shRNA or scrambled cells treated with or without 4 Gy irradiation. HUVECs (5 × 10^4^) were suspended in 500 *μ*L of conditioned medium and then plated onto the polymerized Matrigel and incubated at 37°C for 6 h. The capillary tube-like structures formed by HUVECs were photographed and counted under a phase contrast inverted microscope.

### 2.10. ELISA for VEGF

U87MG and U251 cells transfected with Nox4-shRNA or scrambled shRNA were treated with or without 4 Gy radiation and then were plated in 6-well tissue culture plates at a density of 1 × 10^6^ cells per well and incubated at 37°C. The supernatants were collected 12 h after radiation. VEGF concentration was determined using Quantikine ELISA kits (R&D Systems, MN, USA) according to the manufacturer's instructions.

### 2.11. Statistical Analysis

The results were expressed as the mean ± SD. Differences between the two groups were assessed using a two-tailed *t*-test. A *P* value less than 0.05 was considered statistically significant. Statistical analysis was performed with SPSS 13.0 statistical software (SPSS Inc., Chicago, Illinois).

## 3. Results

### 3.1. Lentivirus-Mediated shRNA Inhibited Nox4 mRNA and Protein Expression in GBM Cell Lines

**Figure 1:**
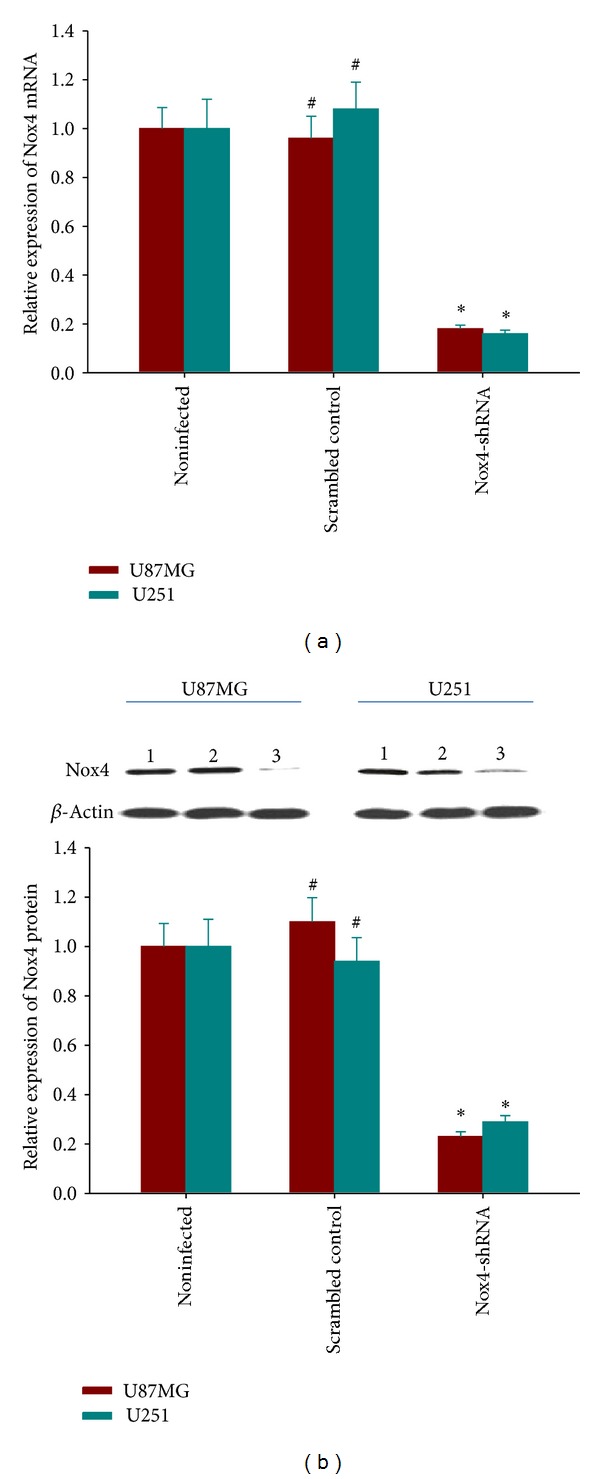
Verification of knockdown of Nox4 expression in U87MG and U251 cells by lentivirus-mediated RNA interference. (a) The expression levels of Nox4 mRNA were measured by qRT-PCR. There was a dramatic decrease of Nox4 mRNA in the Nox4 shRNA group (*P* < 0.05). No significant difference was observed between noninfected cells and scrambled control. (b) The expression levels of Nox4 protein were measured by Western blot (line 1, noninfected; line 2, scrambled control; line 3, Nox4-shRNA). The protein levels in the Nox4-shRNA group decreased significantly compared to scrambled and noninfected cells. The GAPDH gene and the *β*-actin protein are the internal controls for qRT-PCR and Western blot analysis, respectively. Columns, mean; bars, SD; *P < 0.05, versus noninfected and scrambled control; ^#^ *P* > 0.05, versus noninfected.

To investigate the role of Nox4 in GBM, lentivirus vector encoding Nox4 shRNA was constructed and infected U87MG and U251 cell lines. Then, the lentivirus-transduced cells were selected by puromycin for 10 d and the clones stably transfected with pGIPZ-lentiviral shRNAmir (Nox4-shRNA) or pGIPZ nonsilencing control vector (scrambled control) were successfully generated. The positive GFP expression in cells was still above 90% even in these clones cultured up to passage 15. To verify that the Nox4 gene was silenced by the lentivirus vector, the mRNA and protein levels in U87MG and U251 cells were assessed using real-time quantitative PCR and Western blot assays, respectively. Compared with the levels in uninfected and scrambled cells, the Nox4 mRNA and protein levels in U87MG and U251 cells infected with Nox4 shRNA decreased significantly (Figure 1), indicating the successful knockdown of Nox4 in the derived clones.

### 3.2. Nox4 Is Involved in ROS Generation in GBM Cell Lines

**Figure 2:**
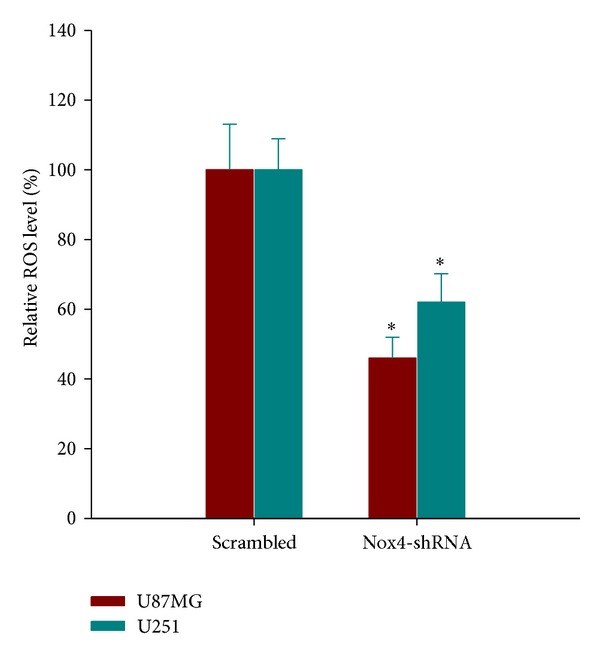
Inhibition of ROS production by Nox4-shRNAs. U87MG and U251 cells transfected with Nox4-shRNA or scrambled shRNA were labeled with DCFH-DA and alterations in the intracellular ROS level were measured by FACS analysis. DCF fluorescence shown in histogram was normalized to that in scrambled cells. The data are means ± SD. *P < 0.05, versus scrambled.

To test whether Nox4 mediates ROS production, intracellular superoxide production was evaluated by using flow cytometry in cells loaded with oxidation-sensitive DCFH-DA. Transfection of Nox4 shRNA resulted in a significant inhibition of ROS production as compared to scrambled controls (Figure 2), suggesting that Nox4 is one of the major sources of ROS generation in U87MG and U251 glioblastoma cells.

### 3.3. Nox4 Silencing Enhanced Radiosensitivity of Glioblastoma Cells

**Figure 3:**
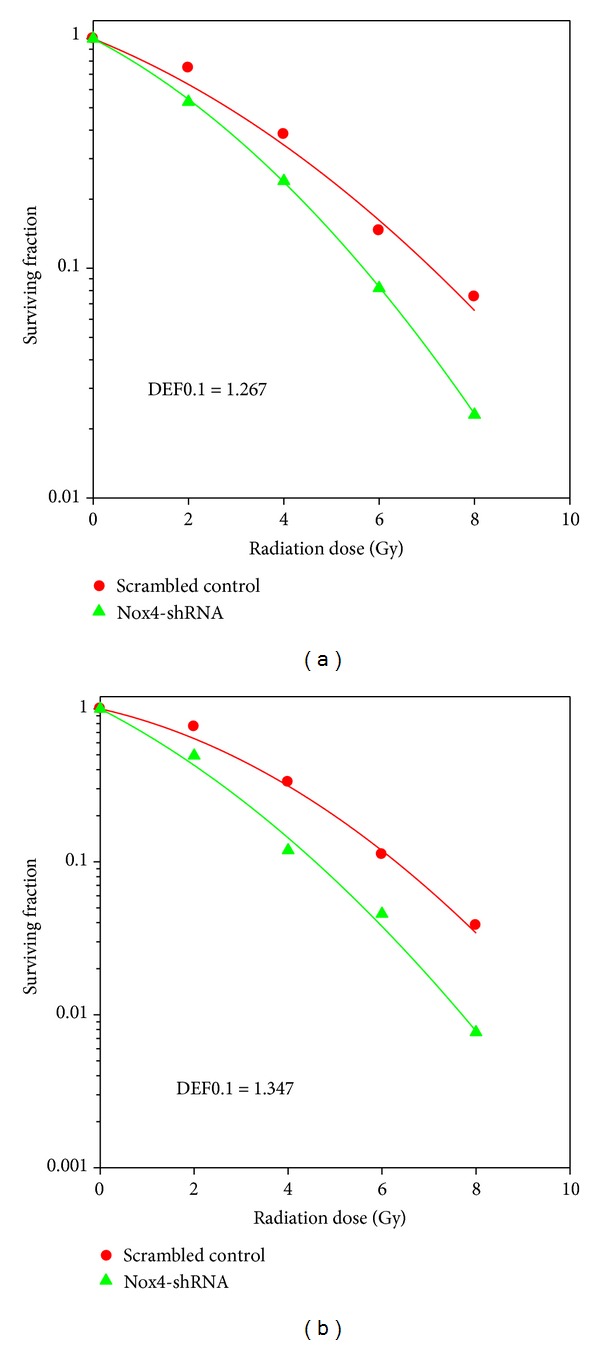
Effect of Nox4 silencing on radiosensitivity of glioblastoma cell lines was measured by clonogenic survival assay. Colony-forming efficiency was determined 10 to 14 d later and survival curves were generated and linear-quadratic (LQ) equation was fitted to data sets. (a) U87MG infected with Nox4-shRNA or scrambled control (DEF0.1 = 1.267 when shRNA versus scrambled control). (b) U251 infected with Nox4-shRNA or scrambled control (DEF0.1 = 1.347 when shRNA versus scrambled control). Points, mean; DEF, dose enhancement factor.

To determine the effect of Nox4 silencing on GBM tumor cell radiosensitivity, clonogenic survival analysis was performed with U87MG and U251 stably transfected with Nox4 shRNA or scrambled control. As shown in Figure 3, Nox4 shRNA caused a significant reduction in clonogenic survival in cell cultures of both U87MGMG (left) and U251 (right) following radiation compared with that caused by scrambled shRNA combined with radiation, resulting in an increase in the radiosensitivity with a dose enhancement factor of 1.267 and 1.347 at a surviving fraction of 10%, respectively.

### 3.4. Nox4 Silencing Suppressed Glioblastoma Cell Proliferation

**Figure 4:**
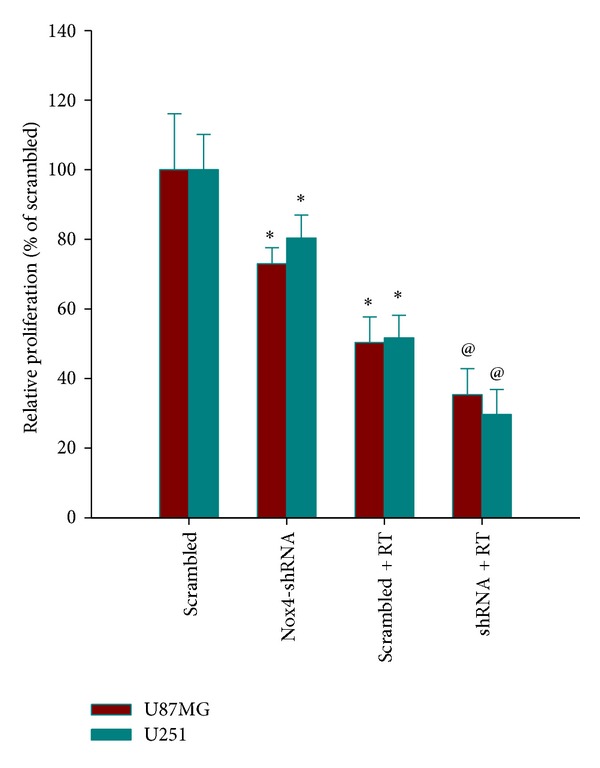
Effect of Nox4 silencing on the proliferation activity of GBM. Proliferation of U87MG and U251 cells transfected with Nox4-shRNA or scrambled shRNA was determined by cell count after 72 h exposure to 4 Gy irradiation. Relative numbers of cells are shown as histogram. Columns, mean; bars, SD; RT, radiation therapy; *P < 0.05, versus scrambled; ^@^ *P* < 0.05, versus scrambled and Nox4-shRNA and scrambled + RT.

To investigate the effect of Nox4 silencing on the proliferation activity of GBM, a cell counting proliferation assay was performed. As shown in Figure 4, Nox4 shRNA transduced cells showed significantly reduced proliferation when compared with the scrambled control group (*P* < 0.05). Radiation treatment also inhibited the cell proliferation (*P* < 0.05). When radiation treatment was combined with Nox4 knockdown, a further reduction of the cell count was observed (*P* < 0.05).

### 3.5. Nox4 Silencing Inhibited Glioblastoma Cell Invasion

**Figure 5:**
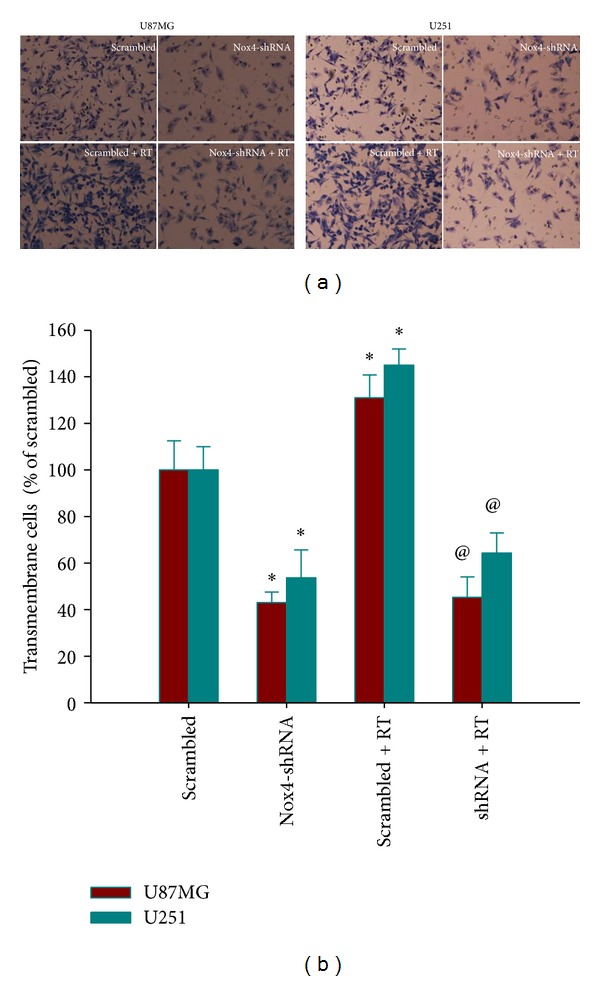
Effect of Nox4 knockdown on cell invasion in GBM. Matrigel invasion assays were performed. The numbers of invading cells were determined by counting the cells stained with 0.01% crystal violet solution in the lower side of the membrane. (a) Stained cells seen under a microscope (200x). (b) The number of transmembrane cells in each group. Columns, mean; bars, SD; RT, radiation therapy; *P < 0.05, versus scrambled; ^@^ *P* < 0.05 versus scrambled and scrambled + RT.

To evaluate the effect of Nox4 knockdown on invasion capacity of GBM cells, transwell system was utilized. As shown in Figure 5, Nox4 shRNA infected cells revealed a pronounced reduction in invasiveness when compared with the scrambled control group (*P* < 0.05). However, irradiation could markedly enhance invasion capability of both U87MG and U251 scrambled cells, which could be counteracted by knockdown of Nox4 (*P* < 0.05). These data suggested that Nox4 silencing could inhibit both constitutive and radiation-induced invasion of GBM cells.

### 3.6. Nox4 Silencing Inhibited Endothelial Cells Tube-Like Structure Formation* In Vitro*

**Figure 6:**
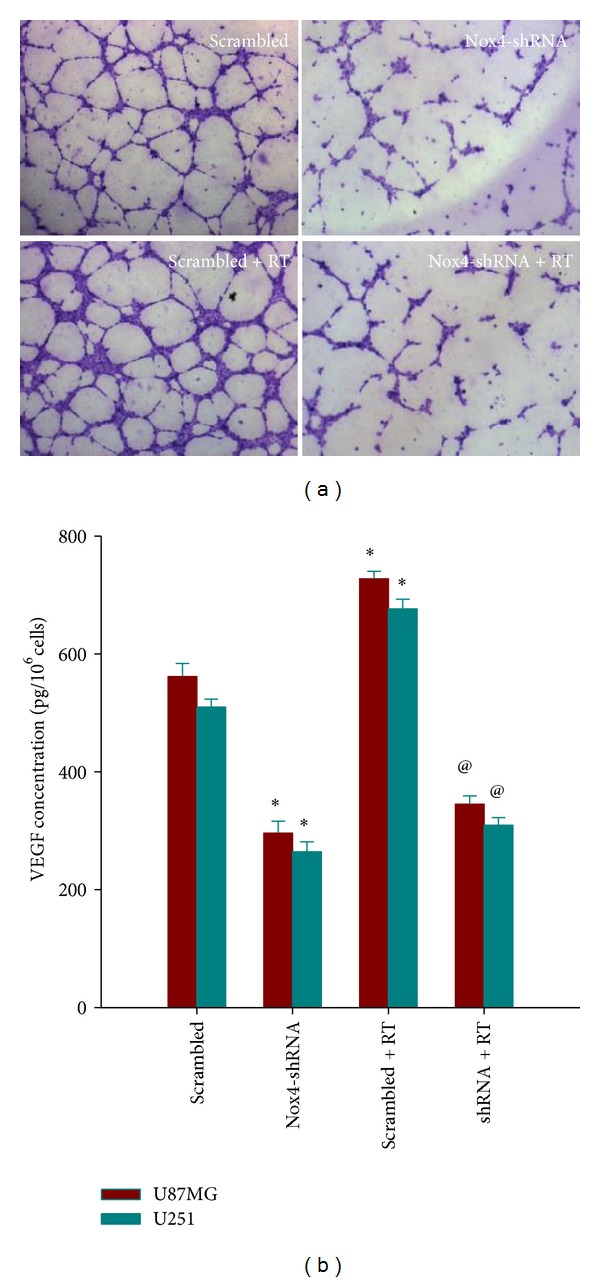
Nox4 silencing inhibited tube-like structure formation of endothelial cells and reduced VEGF expression in glioblastoma cells. (a) Tube-like structure formation ability of human umbilical veins endothelial cells (HUVECs) that were cocultured with conditioned medium derived from Nox4-shRNA or scrambled U87 cells, treated with or without 4 Gy irradiation, was measured. After incubation, endothelial cells were fixed, and tube-like structures were photographed (magnification, ×100). (b) Effects of Nox4-shRNA on expression of VEGF in U87MG and U251 cells. Cells were infected with Nox4-shRNA or scrambled control and treated with or without 4 Gy irradiation. Samples were collected at 24 h postradiation. VEGF protein levels in the culture supernatant were determined by ELISA. Columns, mean; bars, SD; RT, radiation therapy; *P < 0.05, versus scrambled; ^@^ *P* < 0.05, versus scrambled and scrambled + RT.

The sprouting of endothelial cells and formation of tubes are crucial steps in the angiogenic process. To determine the effect of Nox4 knockdown on angiogenesis, we examined how Nox4 shRNA regulates tube-like structures formation of HUVECs* in vitro* using a coculture system. As shown in Figure 6(a), the tube-like structure formation was significantly suppressed by the conditioned medium (CM) from U87MG cells infected with Nox4 shRNA, compared with the CM from scrambled control cells. Moreover, CM from irradiated U87MG cells increased HUVECs tube-like structure formation compared to nonirradiated conditioned medium, and this kind of irradiation-induced tube formation was obvious inhibited by Nox4 knockdown.

### 3.7. Nox4 Silencing Reduced VEGF Expression in Glioblastoma Cells

To determine if Nox4 knockdown could inhibit VEGF secretion, an ELISA assay was used to assess secreted VEGF levels in Nox4 shRNA or scrambled control treated with or without irradiation. These data are summarized in Figure 6(b); the secreted VEGF level of Nox4 shRNA infected cells was much lower than that of scrambled control group (*P* < 0.05). Moreover, irradiation could increase the VEGF level of GBM cells infected with scrambled control, and Nox4 shRNA attenuated the radiation-induced VEGF expression (*P* < 0.05).

## 4. Discussion

The clinical prognosis for glioblastoma patients is extremely dismal [17]. New strategies to treat this deadly disease are desperately needed. In the present study we showed that lentivirus-mediated shRNA silencing of Nox4 can decrease intracellular ROS production, leading to the inhibition of clonogenicity, proliferation and invasion of human GBM cells, and enhancement of their radiosensitivity. More importantly, Nox4 knockdown in GBM cells decreased the levels of VEGF expression and tumor induced angiogenesis.

Although ROS have been conventionally thought to be a cause of stress-induced cell death, they may provide tumor cells with survival advantage over normal counterparts [18, 19]. The major therapeutic feature of radiation is the induction of toxic oxidative damage in targeted cancer cells. ROS are generated from cellular water by high-energy deposition during radiation, which oxidize DNA, proteins, and lipids and cause toxic oxidative damage in the cancer cells. However, ROS are formed constantly as byproducts of normal enzymatic metabolic reactions. Thus, to prevent overwhelming oxidative damage, cells maintain a basal redox balance between prooxidative and antioxidative reactions [20]. In some tumor models [13, 21], increased basal levels of ROS could stimulate the activation and the expression of stress molecules and antioxidative enzymes to better cope with the shift in redox balance. Thus, upon radiation these cells become less sensitive to toxic levels of ROS and resistant to radiation therapy. As shown in our study, one of the major sources of ROS generation in U87MG and U251 cells is the Nox4 (Figure 2). Accordingly, inhibition of Nox4 using lentivirus-mediated Nox4 shRNA suppressed glioblastoma cell proliferation and clonogenic survival following radiation (Figures 3 and 4). In this context, our findings provide further evidence for prosurvival activity of ROS and support the view that ROS are important intracellular signaling molecules regulating the balance between survival and cell death.

Local invasive growth is a key feature of glioblastoma, and the high invasion/migration character is considered to be a major therapeutic obstacle for glioblastoma treatment. A number of signaling pathways can be constitutively activated in migrating glioma cells, rendering these cells resistant to cytotoxic insults [22, 23]. Several studies have suggested a close relationship between ROS and tumor cell invasion and metastasis [24, 25]. ROS can serve as signaling molecules or can directly oxidize important cellular proteins. The signaling pathways that are associated with ROS include MAPK, PI3K, Rho-GTPase, and NF-*κ*B. ROS can regulate cell adhesion pattern and activate GTPase that promotes cellular migration and invasion through upregulating matrix metalloproteinase (MMPS) or inhibiting tissue inhibitors of metalloproteinases (TIMPS) [26, 27]. In line with previous reports, our study demonstrated that Nox4 knockdown significantly attenuated glioblastoma tumor cell invasion. Although ionizing radiation is the mainstay of nonsurgical treatment in GBM, radiation may promote migration and invasiveness of glioblastoma cells [28, 29]. In agreement with these findings, our experiment indicated that radiation alone promoted glioblastoma cell invasion while Nox4 silencing using lentivirus-mediated shRNA strikingly suppressed radiation-provoked tumor cell invasion, supporting the critical role of Nox4-generated ROS in glioblastoma cell invasion. The data also rationalized the combination conventional radiotherapy with inhibition of Nox4 system with shRNA or a substance with similar properties to counteract the potential undesired proinvasive effect of radiotherapy.

In addition, activated tumor angiogenesis is another characteristic feature of GBM, contributing to tumor invasiveness and radioresistance. Accordingly, antiangiogenic therapy has been successfully introduced into clinical glioblastoma treatment, for example, via VEGF/VEGFR signaling inhibition [30]. ROS derived from NADPH oxidase have been suggested to play an important role in physiological and pathological angiogenesis [31, 32]. They can function as signaling molecules to mediate various angiogenic-related responses such as cell proliferation, migration, and angiogenic gene expression in ECs and cancer cells. Xia et al. [15] reported that ROS regulated hypoxia-inducible factor 1 (HIF-1) and vascular endothelial growth factor (VEGF) expression in ovarian cancer cells. Elevated levels of endogenous ROS were required for inducing angiogenesis and tumor growth. Nox4 knockdown in ovarian cancer cells decreased the levels of VEGF and HIF-1A and tumor angiogenesis. This notion was corroborated in our study because Nox4 silencing significantly reduced the level of VEGF expression of nonirradiated glioblastoma cells and inhibit endothelial cells tube-like structure formation induced by conditioned medium from nonirradiated U87MG cells. Many studies have reported that radiotherapy can stimulate multiple signal transduction pathways simultaneously and alter the expression of proangiogenic molecules including VEGF in surviving cancer cells and host cells [33, 34]. In the present study, we further confirmed that irradiation induced a marked increase in VEGF protein expression in glioblastoma cells, and Nox4 shRNA could potentially block the radiation-induced enhancement of VEGF. Moreover, Nox4 shRNA was shown to inhibit tube-like structure formation of cocultured endothelial cells induced by X-ray irradiation. Therefore, knockdown of Nox4 suppressed both constitutive and radiation-induced angiogenesis* in vitro*, suggesting an important role of Nox4 derived ROS in angiogenesis process in glioblastoma.

In conclusion, our findings indicate that Nox4 is associated with tumor invasion, angiogenesis, and radioresistance in glioblastoma. Knockdown of Nox4 expression reduced ROS production significantly and suppressed glioblastoma cell proliferation and invasion and tumor induced angiogenesis as well as increased their radiosensitivity. Therefore, inhibition of Nox4 by lentivirus-mediated shRNA may be a strategy to overcome radioresistance and then improve its therapeutic efficacy for glioblastoma.
